# Follow-up of periapical inflammation after root canal retreatment and surgical endodontic treatment using dental-dedicated magnetic resonance imaging: a case report

**DOI:** 10.1007/s11282-026-00948-0

**Published:** 2026-07-02

**Authors:** Jennifer Christensen, Rubens Spin-Neto, Lise-Lotte Kirkevang

**Affiliations:** Section for Oral Radiology and Endodontics, Department of Dentistry and Oral Health, Aarhus University, Vennelyst Boulevard 9, 8000 Aarhus, Denmark

**Keywords:** ddMRI, Apical periodontitis, Diagnosis, Inflammation

## Abstract

The aim is to present a dental-dedicated magnetic resonance imaging (ddMRI)-based diagnosis and follow-up in a clinical case of apical periodontitis (AP) on a root canal treated (RCT) tooth. Persistent AP in RCT teeth can be difficult to distinguish from scar tissue or incomplete healing. In the absence of symptoms, no currently known imaging method can verify the presence of inflammation. ddMRI is a novel imaging modality proposed for dentistry, that has the potential to reveal inflammation. A 50-year-old male presented with mild symptoms from a previously RCT 36. ddMRI suggested mild inflammation, and an intraoral image revealed a periapical radiolucency. The tooth was non-surgically endodontically retreated. During follow-up, no exacerbation of the inflammation was detected on ddMRI. However, one year after retreatment, a periapical radiolucency persisted, and the ddMRI indicated that the inflammation had not resolved. Surgical endodontic retreatment was performed, and a tissue sample was retrieved from the periapical area. A histopathological analysis revealed inflammation as suspected from the hyperintense signal seen on ddMRI. Despite treatment, the inflammation did not resolve, and after development of a fistula, the tooth was extracted.

**Supplementary Information:**

The online version contains supplementary material available at https://doi.org/10.1007/s11282-026-00948-0.

## Introduction

Infection of the dental pulp can lead to inflammatory changes in the periapical bone if not treated [1, 2]. These changes include demineralization of the periapical bone, resulting in a periapical radiolucency as depicted on a radiographic image [1, 2]. Following successful root canal treatment (RCT), the periapical lesion is expected to heal, new bone replacing the inflamed periapical tissue [1, 2]. The healing process of the alveolar bone after RCT is of interest for dentists and patients and is important to assess the possibility for future rehabilitation and function, e.g., if the tooth is to serve as an abutment for fixed prosthetics. The healing process takes place in different stages, that evolve from the decrease in the number of inflammatory cells, to the formation and maturation of collagen, neovascularization and eventually *de novo* bone formation [3]. The process is normally completed after 6–12 months provided that the RCT successfully eliminated the source of the inflammation. However, the amount of mineralization of the periapical lesion is variable, ranging from a more dense and well-organized fibrous connective tissue (i.e., scar) [4, 5] to fully mineralized bone, sometimes even followed by formation of cementum and new periodontal ligament organization. Imaging such processes in dentistry is limited to modalities based on ionizing radiation (i.e., X-rays) which show the amount of mineralized tissue in the area at the moment of imaging. Adding to that, radiographic images cannot differentiate between different kinds of soft tissues, e.g. granulation tissue and scar tissue, which might not be as mineralized as the surrounding bone and therefore appear more radiolucent. Thus, a persisting periapical radiolucency can indicate active inflammation, but can also merely indicate scar tissue. Another diagnostic challenge is that the size of periapical lesions is often underestimated in radiographs, since substantial bone degeneration (i.e., 30–50% mineral loss) is necessary to identify lesions radiographically [6, 7]. Finally, and extremely relevant, radiographic imaging is based on potentially harmful ionizing radiation. Based on the current available methods, clinicians cannot usually distinguish between immature or soft bone healing and persistent active inflammation, which may potentially lead to overtreatment.

Dental-dedicated magnetic resonance imaging (ddMRI) is a modification of traditional MRI, proposed for imaging of the dental tissues [8]. The method is under development and is based on the magnetic properties of the tissues rather than their ability to absorb radiation. This modality might be a suitable alternative within dentistry for treatment planning and follow-up after endodontic treatment. Furthermore, the modality might support the dentist in the assessment of the treatment outcome (i.e., tissue healing). ddMRI introduces the possibility to obtain images of different tissue types, to evaluate bone formation and resolving inflammation without the radiation burden from conventional X-ray based modalities and potentially individualize the monitoring of periapical healing after RCT in humans.

The aim of this case report is to present a ddMRI-based diagnosis and follow-up in a clinical case of apical periodontitis (AP) on a RCT tooth.

## Case report

This case report has been written according to Preferred Reporting Items for Case reports in Endodontics (PRICE) 2020 guidelines [9]. (Online Resource 1).

**Fig. 1:**
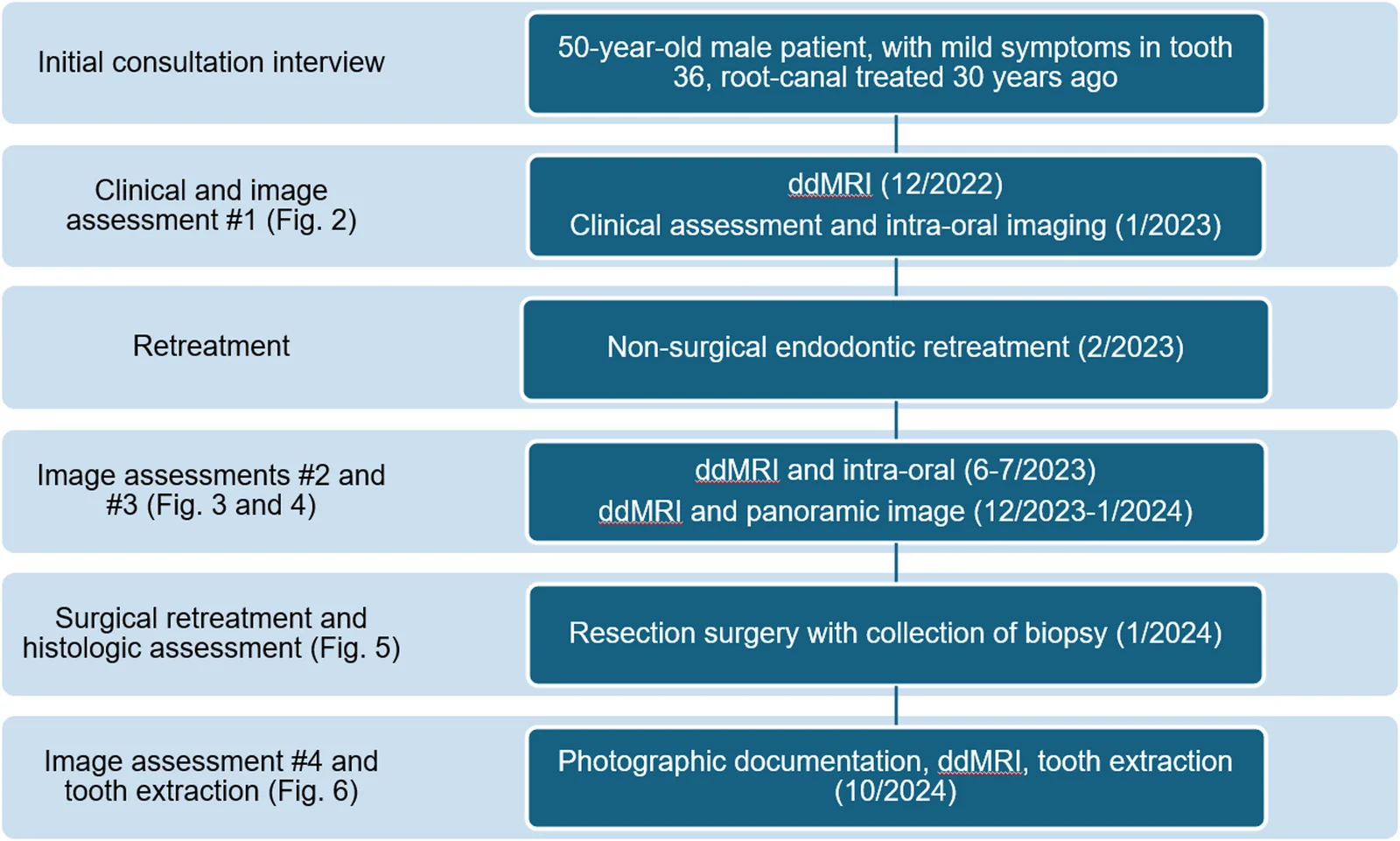
Timeline depicting imaging and treatment steps

**Fig. 2:**
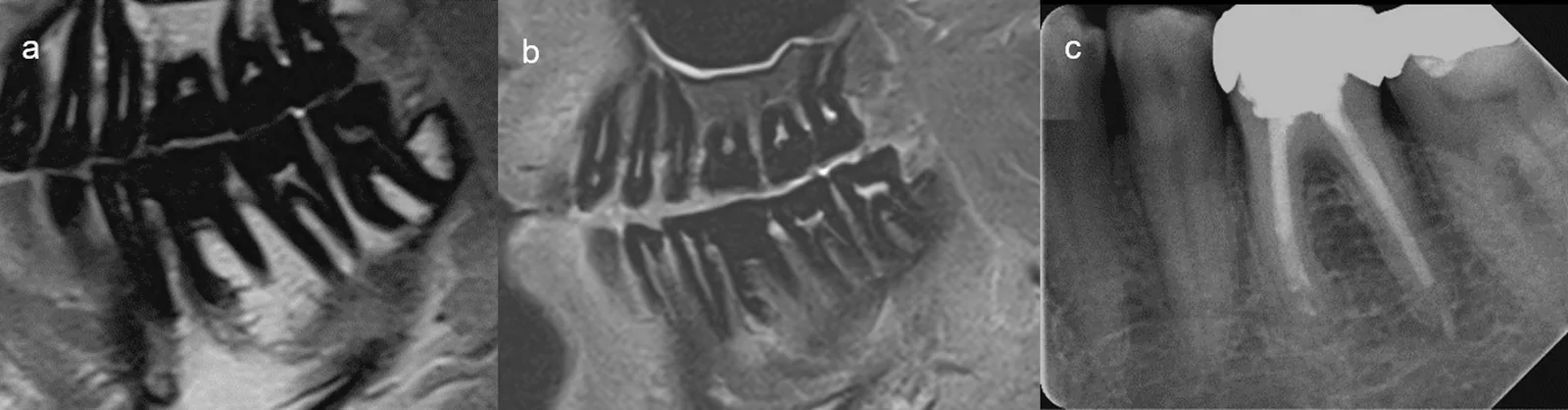
Images acquired before non-surgical endodontic retreatment of 36 (Dec. 2022 and Jan. 2023, respectively). **a** Sagittal ddMRI section (PD) showing tissue alteration at the mesial root of 36. **b** Sagittal ddMRI section (STIR) showing hyperintense signal at the mesial root of 36. **c** Intraoral image showing periapical radiolucency at the mesial root of 36

A 50-year-old male presented in December 2022 with mild recurring symptoms for the past year from the left lower jaw. The timeline of the treatment course is shown in Fig. 1. The patient had no medical conditions and did not take any medication. He was fully dentate (apart from one implant replacing tooth 46), had three RCT teeth and several restorations. Tooth 36 had received RCT in three root canals more than 30 years ago. A ddMRI was performed on Dec 13th, 2022 (Fig. 2a and b) using a Siemens Magnetom Free.Max 0.55T (Siemens Healthineers AG, Forchheim, Germany) with a 7-channel radiofrequency dental-dedicated coil (RAPID Biomedical GmbH, Rimpar, Germany). The imaging sequences used are displayed in Table 1 and were repeated for subsequent ddMR imaging. The images were assessed by the three authors, all with experience in ddMRI, in an office with dimmed light, using dedicated software to navigate the image sections, adjust the windowing, and also providing possibilities for zooming into the areas of interest area (RadiAnt, Medixant, Poznan, Poland).

**Table 1:** Available pulse-sequences used in the ddMRI examinations

Application	Basic description	Resolution (mm)	FOV (h × w × d ; mm)	Acquisition time (min‘s“)	Basic scan parameters
Anatomy longitudinal	PD (TSE)	0.3 × 0.3 × 2.0	263 × 350 × 350	2‘28“	TR 2400.0 ms, TE 50.00 ms, FA1 150°, FA2 90°, bandwidth 100 Hz/pixel, GRAPPA acceleration (x3), averages 4, encoding A-P
Anatomy cross-sectional	PD (TSE)	0.3 × 0.3 × 2.0	263 × 350 × 350	2‘28“	TR 2400.0 ms, TE 48.00 ms, FA1 150°, FA2 90°, bandwidth 100 Hz/pixel, GRAPPA acceleration (x3), averages 4, encoding R-L
Inflammation longitudinal	PD (STIR)	0.4 × 0.4 × 2.0	263 × 350 × 350	2‘38“	TR 2560.0 ms, TE 46.00 ms, FA1 130°, FA2 90°, bandwidth 106 Hz/pixel, GRAPPA acceleration (x3), averages 6, encoding A-P
Inflammation cross-sectional	PD (STIR)	0.4 × 0.4 × 2.0	263 × 350 × 350	2‘57“	TR 2310.0 ms, TE 46.00 ms, FA1 130°, FA2 90°, bandwidth 106 Hz/pixel, GRAPPA acceleration (x2), averages 5, encoding R-L

*FA* flip angle, *FOV* Field of View, *GRAPPA* generalised auto-calibrating partially parallel acquisition, *Hz* Hertz, *PD* proton density, *ms* milliseconds, *STIR* short-tau inversion recovery, *TE* time to echo, *TR* repetition time, *TSE* turbo spin-echo

The exams always started with a scout acquisition of approximately 1 min, based on which the next pulse sequences were planned. The pulse sequences are presented as originally named by the producer, according to what will typically be highlighted in these sequences (“Anatomy” and “Inflammation”), therefore, it is important to note that a sequence named “inflammation” does not necessarily emphasize “inflamed tissue.”

The images showed a hyperintense area around the apex of the mesial root of the tooth 36, suggesting mild active inflammation and tissue alteration. Informed, valid consent was obtained from the patient for further investigation.

In January 2023, the patient consulted his regular dentist, who performed an intra-oral radiographic examination and found a periapical radiolucency at the mesial root (Fig. 2c). The patient was referred to a specialist for non-surgical endodontic retreatment of tooth 36.

### Non-surgical endodontic retreatment on February 6th, 2023

Non-surgical endodontic retreatment was performed using aseptic techniques and operating microscope. After applying rubber dam, access to the root canal system was obtained, and the existing root filling was removed with Protaper Ultimate (TM Dentsply Sirona, NC, USA). Removal of the root filling material proved to be difficult since the apical part of the root canals were widened and irregular. During the retreatment an untreated disto-buccal canal was detected and treated. Irrigation with 3.0% sodium hypochloride, applying ultrasonic (Newtron Acteon, Norfolk England) and sonic (Endo-Activator Dentsply Sirona, NC, USA) activation, was performed, followed by irrigation with EDTA. The root canals were dried with paperpoints and obturated with gutta-percha (ProTaper Ultimate Conform Fit Gutta-percha / Dentsply Sirona, NC, USA) and bioceramic sealer (Total Fill BC-Sealer/FKG, Le Crêt-du-Locle, Switzerland). The tooth was coronally restored with composite (Syntac and Tetric, Ivoclar Vivadent AG, Schaan, Liechtenstein).

**Fig. 3:**
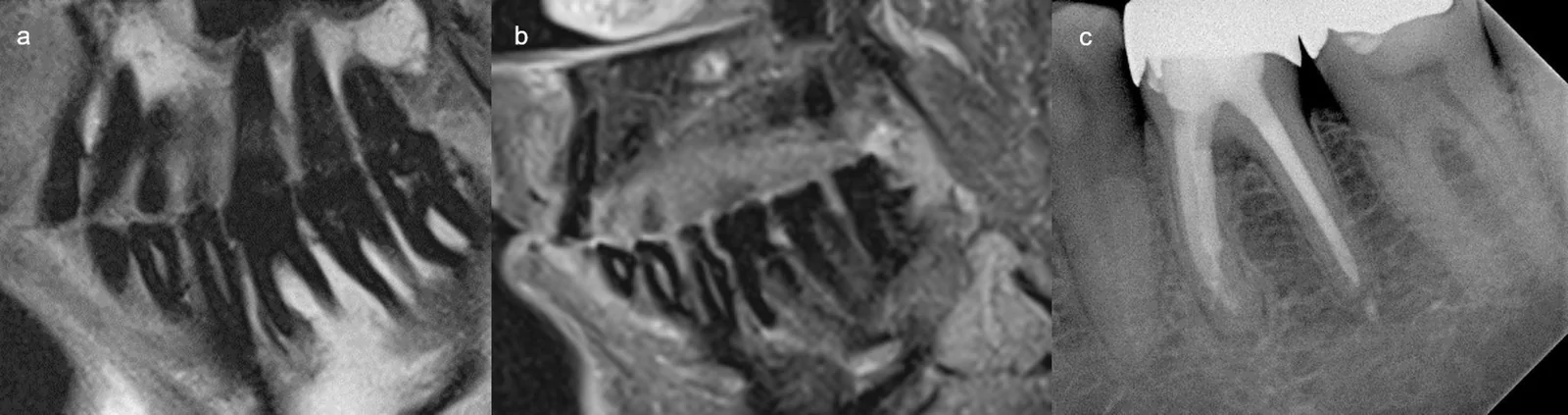
Images acquired approximately 6 months after non-surgical endodontic retreatment of 36 (June 2023 and July 2023, respectively). **a** Sagittal ddMRI section (PD) showing tissue alteration at the mesial root of 36. **b** Sagittal ddMRI section (STIR) showing hyperintense signal at the mesial root of 36. **c** Intraoral image showing periapical radiolucency at the mesial root of 36

After retreatment, the patient had six follow-up ddMR images over a period of six months, with the final follow-up on June 26th 2023 (Fig. 3a and b). The images suggested no progression of the lesion, in some images even healing could be implied, though without providing absolute information on the completion of the healing. The 6-month post-treatment control intraoral image in July 2023 (Fig. 3c) showed little change compared to the immediate post-treatment image.

**Fig. 4:**
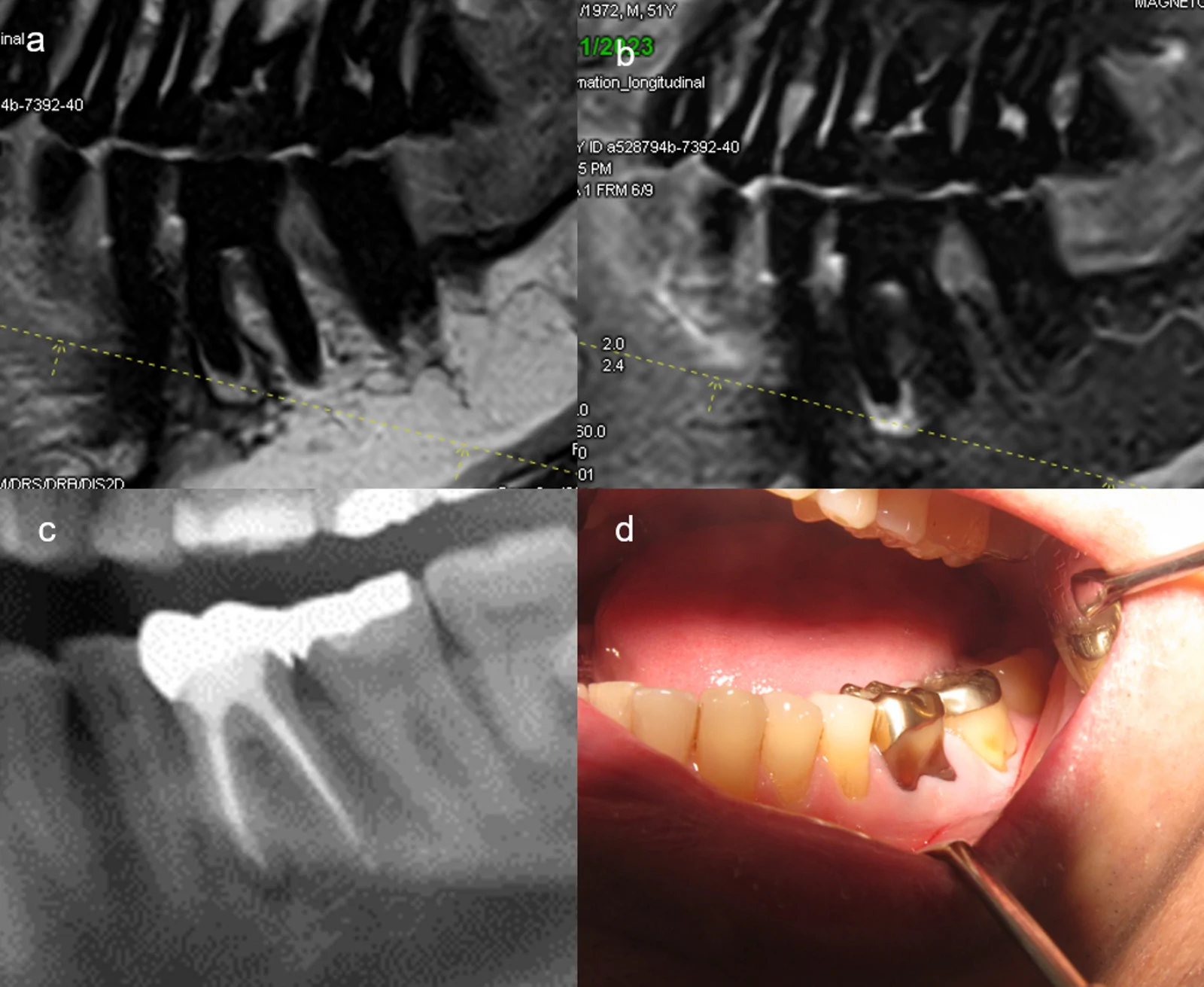
Images acquired approximately 11 months after non-surgical endodontic retreatment of 36 (Dec. 2023 and Jan. 2024, respectively). **a** Sagittal ddMRI section (PD) showing exacerbated tissue alteration at the mesial root of 36. **b** Sagittal ddMRI section (STIR) showing increased hyperintense signal at the mesial root of 36. **c** Section of panoramic image showing periapical radiolucency at the mesial root of 36. **d** Clinical photo showing retraction of the marginal soft tissue, but otherwise healthy conditions around 36

It was decided to perform a follow-up ddMRI again five months later. ddMRI acquired on December 21st, 2023 (Fig. 4a and b) revealed remaining hyperintense signal around the mesial apex, suggesting that the inflammatory process had exacerbated. This was confirmed on a panoramic image in January 2024 (Fig. 4c). It was then decided to perform surgical endodontic retreatment.

## Surgical endodontic retreatment January 9th, 2024

**Fig. 5:**
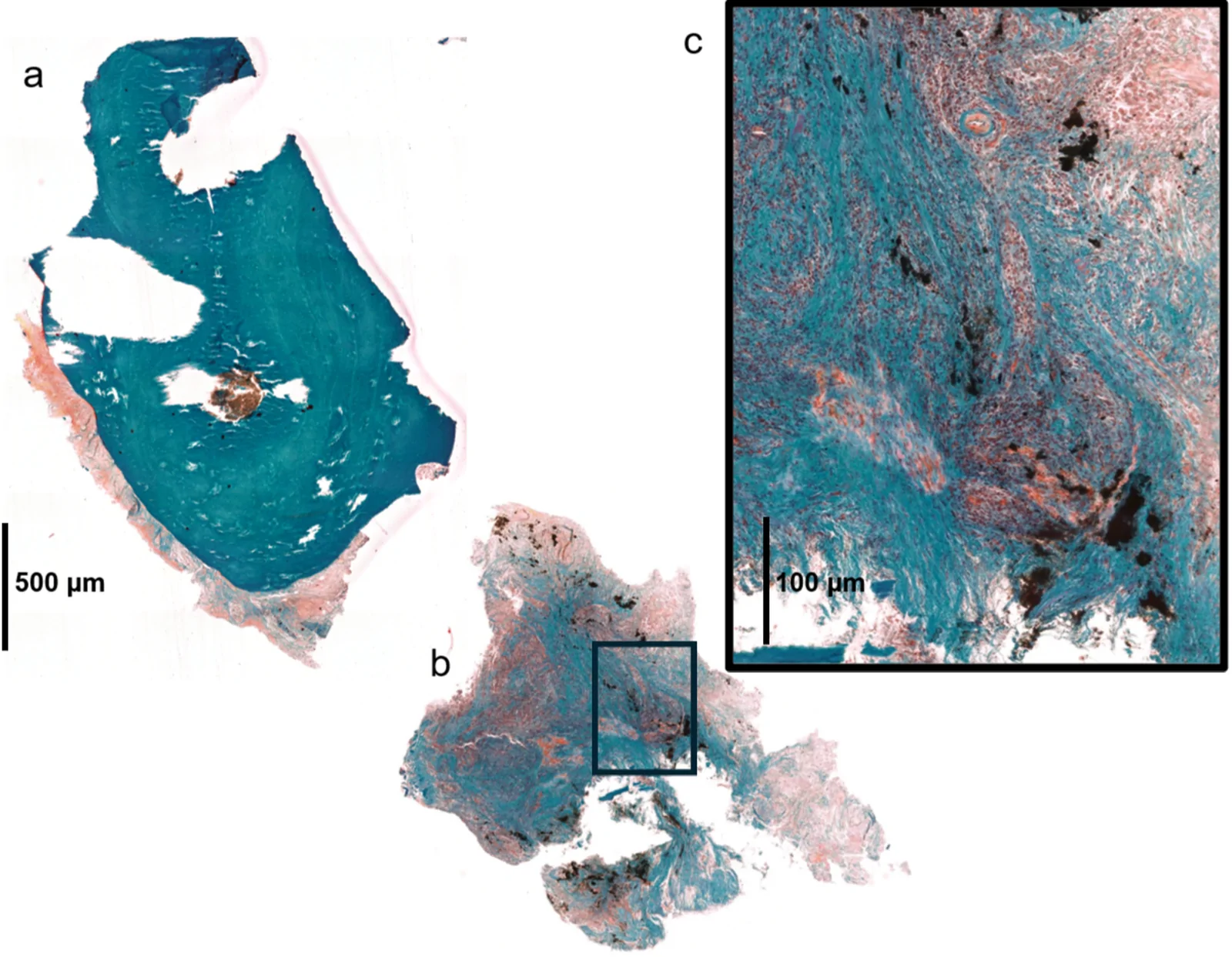
Histopathological sections (Goldner’s trichrome staining) depicting the apical tissues (**a**, dentin; **b**, periapical lesion) biopsy, in which both a granulation tissue and fibrous tissue are visible

The root tip of the mesial root of tooth 36 was resected, microsurgical ultrasonic preparation of the two root canals was performed, and filled with IRM (Dentsply Sirona, NC, USA). A histologic sample was taken from the apical area and sent to histopathological examination. The results from this examination confirmed the presence of active inflammation in the tissue (Fig. 5).

After the surgical endodontic retreatment, the patient had follow-up ddMR images on three occasions during 2024 (Jan 19th, March 7th, July 3rd ). These images suggested lack of healing and persistent active inflammation in the area.

**Fig. 6:**
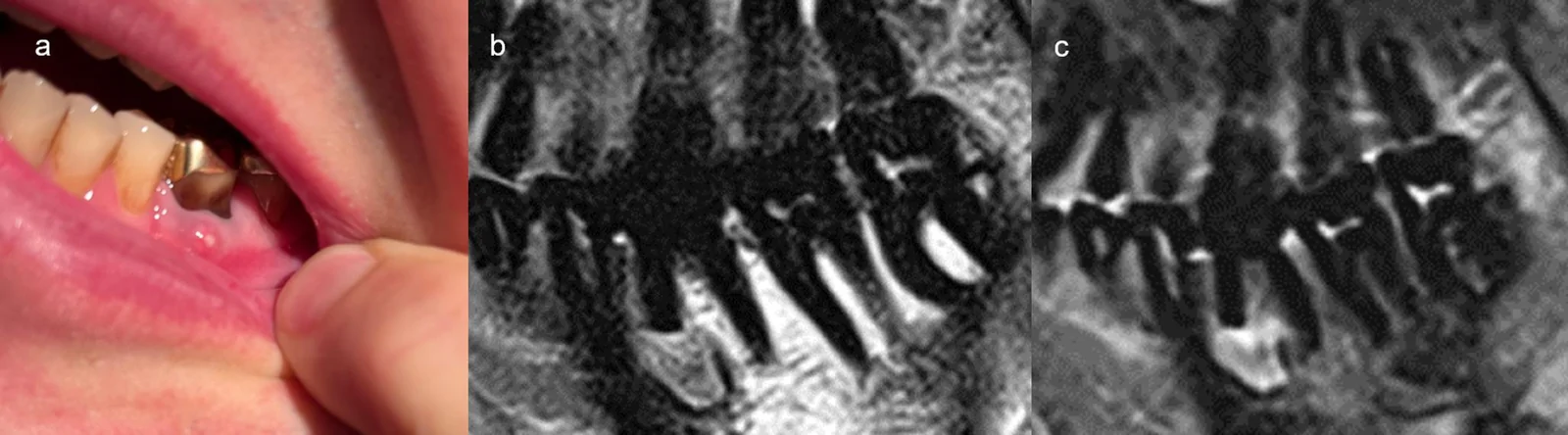
Images acquired approximately 9 months after surgical endodontic retreatment of 36 (Oct 2024). **a** Clinical photo showing fistula buccal to 36. **b** Sagittal ddMRI section (PD) showing tissue alteration at the mesial root of 36. **c** Sagittal ddMRI section (STIR) showing increased hyperintense signal at the mesial root of 36

At the clinical follow-up examination (October 24th, 2024), a fistula in the vestibular region of tooth 36 was seen, as well as soft tissue recession and bone loss in the cervical area (Fig. 6a). A final ddMRI (October 25th, 2024) (Fig. 6b and c) was acquired and confirmed persistent active inflammation. The decision was made to extract the tooth. After extraction, (October 30th, 2024) the tooth was inspected and a vertical root fracture in the mesial root was detected. However, it was not possible to define if the fracture was induced during extraction, or if it was already present and possibly causing the continuation of the disease, even after endodontic treatment.

## Discussion

ddMRI is a new modality proposed for dentomaxillofacial imaging [8]. The possible added value of ddMRI over those imaging modalities traditionally used in dentistry comes from the possibility of depicting the soft tissues of the patient in detail, without the radiation burden associated with panoramic imaging or CBCT [10]. For all new technologies, the establishment of the evidence through the evidence hierarchy proposed by Fryback and Thornbury (F&T) should be upheld [11]. Currently, some preliminary evidence, including studies on F&T level 1 have been performed showing that the technical quality of the ddMR images is sufficient to detect hard and soft tissue alterations in the dentomaxillofacial region, from the diagnosis to the follow up stage [8, 10, 12–14]. The obvious next step is to assess the diagnostic accuracy of the new imaging technology for specific diagnostic tasks, such as apical periodontitis (AP). As this information is not yet available, for the present case report the clinical signs and symptoms, allied to traditional imaging (i.e., periapical images) were always the basis for decision making. ddMRI was done additionally to see if the findings from the ddMRI aligned with the clinical and radiographic findings, and if it had the potential to add valuable information to be used in the decision-making process. Here it becomes essential to know the true state of the tissue to provide a solid reference standard [11]. To our knowledge, no level 2 studies focusing on endodontic disease have yet been performed. The appropriate reference standard for diagnosing AP is histopathological examination of the periapical area. Thus, for ddMRI it is important to verify findings made on ddMR images against histological findings to confirm if what is perceived as inflammation when assessing the images, is truly active inflammation, and not merely healthy fibrous periapical tissue.

To our knowledge the presented case is the first case report where ddMRI has been utilized as an additional diagnostic tool to guide endodontic decision-making. Furthermore, it is the first case where an endodontic diagnosis based on the combination of clinical information, radiographic information and ddMRI has been verified by histology.

In the present case endodontic retreatment was indicated due to radiographic signs of persistent active inflammation in the periapical area, in addition to the patient’s complaint of mild recurring symptoms from the area. However, the literature is very straight-forward defining that even the most advanced CBCT possibilities are not reliable as gold standard when it comes to depicting the actual content of a periapical radiolucent area, and over- / under-interpretation of disease activity are common [15]. In ddMR images a hyperintense signal in the periapical area was seen and interpreted as a sign of active inflammation, contrary to the presence of artefacts, or to a “scar” area, that would be of intermediate or even hypointense in the images. A wound healing process would mimic the appearance of inflammation due to the increased blood flow and cell concentration, resulting in a hyperintense signal. The same applies to other conditions that present with excess fluid. Thus, differentiating between these conditions could potentially be difficult. However, it was later demonstrated that the hyperintense signal in the periapical area detected on ddMRI before the surgical endodontic retreatment could be confirmed as active inflammation by histologic examination (i.e., gold standard for disease definition). The same hyperintense signal had been detected before the non-surgical endodontic retreatment and again at the follow-up after surgery, in which cases the same histological result would be expected. One could speculate if an undiagnosed vertical root fracture could have been the cause of the persistence of the disease. In that direction, at the time of the study, ddMRI was not yet tailored to include the detection of root fractures, and as the other imaging modalities and clinical assessment did not point at the direction, this condition was not assessed prior to tooth extraction. With that in mind, it is not possible to ensure the fracture was present before, or was induced during the extraction procedure.

In many cases patients do not experience any symptoms even though radiographic information indicates that a periapical lesion is present. In non-root filled cases, clinical vitality testing can be performed to reach a correct diagnosis, but in root filled teeth we have relied on radiographic information, even though studies have shown that there is an increased risk of overdiagnosing [15]. In these cases, ddMRI may prove to be an additional diagnostic tool because it can potentially allow differentiation between active inflammation and scar tissue/incomplete healing. One must be aware, though, that wound healing also will present as a radiolucent and hyperintense area on radiographs and ddMRI, respectively, and therefore differentiating between persistent inflammation and ongoing healing in some cases could prove to be difficult even with ddMRI [14]. Other conditions such as myeloma or leukemia must also be considered in the case of hyperintense signal without any clinical signs of inflammation [16, 17].

AP is a prevalent condition worldwide and is most prevalent in root filled teeth [18]. It has been suggested that AP in non-root filled teeth may affect the general health more than AP in root filled teeth [19]. However, it is still not known to what extent presence of AP may affect our general health, even though studies indicate that some systemic diseases may be associated with endodontic disease [20]. This may in turn suggest that having even low-grade inflammation may be more problematic for people with general health problems which indicate that they may be in high risk of severe disease courses [19]. In addition, currently patients have teeth extracted to minimize risk of infection e.g. prior to heart surgery, even though the evidence for this practice is sparse, and despite the risk that the dental procedure may pose a risk to the patient [21]. It would be beneficial both to the patient and to society to have a non-invasive risk assessment tool that could distinguish between inflamed and non-inflamed teeth and thereby determine how many radiologically apparent APs are actually inflammatory lesions, and how many are merely scar tissue.

It is important to keep in mind that currently, treatment planning takes place based on present knowledge, guidelines, and “best practice”. For the time being, the observation of a tooth with a periapical radiolucency may still be relevant, provided the patient does not experience symptoms and is not a high-risk patient. The literature reports cases that display periapical healing as long as 20 years after treatment [22]. This case indicates that ddMRI might be useful in guiding decision making regarding endodontic retreatment, but future studies will be needed to verify this. A relevant aspect to consider is operator training, when it comes to assessing ddMR images. As this is a novel modality, the operators must also follow a cautious approach before defining a tooth as diseased and not determine a treatment plan solely based on these images. As usual, the clinical findings should north the treatment plan, assisted by the traditional imaging modalities, while evidence on the accuracy of ddMRI for this specific diagnostic task grows.

This case study suggests that ddMRI can be used to detect active periapical inflammation. This is, however, limited to a single case, and it cannot form the basis for clinical recommendations. More cases and clinical studies are warranted, and until these studies have been performed, one should use and interpret the ddMR images with caution.

## Supplementary Information

Below is the link to the electronic supplementary material.

Supplementary Material 1
