# Supplementary material for: Follow-up of periapical inflammation after root canal retreatment and surgical endodontic treatment using dental-dedicated magnetic resonance imaging: a case report
Source: Oral Radiol. 2026 Jul 2;42(4):1287–94. doi: 10.1007/s11282-026-00948-0 (PMC13619786; doi:10.1007/s11282-026-00948-0)
Supplement: Supplementary file 1 [file 11282_2026_948_MOESM1_ESM.pdf]

PRICE 2020 Flowchart

Male patient, age 50

↓

Mild recurring symptoms from lower left jaw

↓

Verbal informed valid consent to further examination and treatment

↓

No medication

↓

Tooth 36 RCT >30 years previously

↓

No relevant clinical findings

↓

ddMRI showed tissue alterations and hyperintense signal at mesial root 36. Intraoral image showed periapical radiolucency at mesial root 36

↓

Differential diagnosis: Incomplete healing after RCT 36

↓

Persistent AP 36

↓

Options: surgical or non-surgical retreatment

↓

Informed valid consent for treatment

↓

Non-surgical endodontic retreatment 36

↓

Follow-up at 6 months and 1 year

↓

Follow-up methods: ddMRI and radiographic imaging

↓

Treatment outcome: Persistent AP, followed by surgical retreatment. Final outcome: Extraction of 36

↓

Patient perspective: N/A

↓

Conclusions: ddMRI may be beneficial in diagnosing apical pathology

↓

No funding or conflict of interest

***From: Nagendrababu V, Chong BS, McCabe P, Shah PK, Priya E, Jayaraman J, Pulikkotil SJ, Setzer FC, Sunde PT, Dummer PMH (2020) PRICE 2020 Guidelines for reporting case reports in Endodontics: A consensus-based development. *International Endodontic Journal* doi: 10.1111/iej.13285.**

For further details visit: http://pride-endodonticguidelines.org/price/
